# EXPLORING LINKS BETWEEN COGNITIVE AGING AND FEATURES OF WORKING MEMORY VIA COMPUTATIONAL MODELING

**DOI:** 10.1093/geroni/igad104.2622

**Published:** 2023-12-21

**Authors:** Sharon H Kim, Yanling Li, Karra Harrington, Jonathan Hakun, Daniel Elbich, Martin Sliwinski, Zita Oravecz

**Affiliations:** The Pennsylvania State University, University Park, Pennsylvania, United States; The Pennsylvania State University, University Park, Pennsylvania, United States; The Pennsylvania State University, University Park, Pennsylvania, United States; Pennsylvania State University, State College, Pennsylvania, United States; The Pennsylvania State University, Hershey, Pennsylvania, United States; Pennsylvania State University, State College, Pennsylvania, United States; Pennsylvania State University, State College, Pennsylvania, United States

## Abstract

With an aging population, understanding cognitive aging is essential for developing interventions to support independence and well-being. Additionally, there is a need for sensitive measures that can differentiate subtle cognitive decline related to neurodegenerative disease from age-related changes. In particular, Alzheimer’s Disease and Related Dementia (ADRD) increases in prevalence with age and involves progressive cognitive decline. The Color Shapes task (CST) is a visual working memory task that has been shown to be sensitive to early indicators for ADRD. CST involves feature binding (e.g., mental representation of features into a unified whole) that is impaired in people with ADRD. In an experiment, we manipulated different CST properties to study whether drift diffusion modeling (DDM) can account for individual differences in latent decision-making processes generating manifest CST performance scores. DDM allows us to quantify theoretically meaningful cognitive parameters that underlie task performance captured in reaction times and accuracies. 68 U.S. community-residing adults (28-80 years) completed daily smartphone assessments across 8 days with experimentally manipulated versions of the CST. We implemented a single-step Bayesian estimation of experimental conditions contrasts, latent DDM parameters, and their associations. We show how individual differences in experimental contrasts were related to age in terms of cognitive (drift rate), non-cognitive (non-decision time), and meta-cognitive (boundary separation) parameters. The results highlight the benefits of using cognitive process models with optimized cognitive tasks to delineate features of cognitive aging for ADRD research.

